# Changes of choroidal thickness and choroidal vascularity index in patients with macular edema secondary to branch retinal vein occlusion after intravitreal ranibizumab

**DOI:** 10.1186/s40942-025-00727-9

**Published:** 2025-09-26

**Authors:** Yuanyuan Qi, Daniel Hillarion Scotland, Chao Zhang, Jiayang Xu

**Affiliations:** 1 Department of Ophthalmology, The Third People’s Hospital of Dalian University of Technology, Dalian, China; 2 Key Laboratory of Corneal and Ocular Surface Diseases Research of Liaoning Province, Dalian, China; 3 Liaoning Province Branch Center of National Clinical Research Center for Eye Diseases, Dalian, China; 4 Dalian Medical University, Dalian, 116033 China

**Keywords:** Branch retinal vein occlusion, Choroidal thickness, Choroidal vascularity index, Ranibizumab, Anti-vascular endothelial growth factor

## Abstract

**Background:**

To observe the changes of choroidal thickness (CT) and choroidal vascularity index (CVI) in patients with macular edema secondary to branch retinal vein occlusion (BRVO) after multiple intravitreal injections of ranibizumab.

**Methods:**

A retrospective cohort study was conducted on 91 patients (91 eyes) with unilateral BRVO treated with a 3 + PRN (pro re nata) regimen of ranibizumab from January 2022 to March 2023. Optical coherence tomography (OCT) was used to measure central retinal thickness (CRT). Enhanced depth imaging OCT (EDI-OCT) was used to measure subfoveal CT (SFCT), nasal CT (1.5 mm from the fovea), and temporal CT (1.5 mm from the fovea) to calculate the mean CT. Choroidal images were binarized using ImageJ software to quantify the luminal area (LA), stromal area (SA), and total choroidal area (TCA), from which CVI (LA/TCA) was calculated. These parameters were evaluated at baseline and 1 month after each injection and were compared across different types of macular edema and between the acute and stable phases of BRVO.

**Results:**

At baseline, the cystoid macular edema (CME) group had significantly lower CRT and SFCT compared to the diffuse retinal thickening (DRT) and mixed-type groups (*P* < 0.01); however, best-corrected visual acuity (BCVA) and CVI did not differ significantly among the groups. In BRVO-affected eyes, CT, LA, SA, TCA, and CVI were all significantly higher than in contralateral eyes (*P* < 0.01). Compared to baseline, CT decreased significantly after the first injection and stabilized after the second (*P* < 0.01). CVI decreased significantly after the second injection and remained stable thereafter (*P* < 0.01). These changes persisted for at least six months after the final injection.

**Conclusions:**

BRVO affects both retinal and choroidal structures. BRVO-affected eyes exhibit choroidal vasodilation, stromal thickening, and have higher CT and CVI values compared to unaffected eyes. Anti-VEGF therapy effectively reduces CT and CVI during the acute phase, leading to a stable state. CVI values do not appear to differ based on the morphological type of macular edema.

**Trial registration:**

ChiCTR2400090054. Registered on 13 November 2023, retrospectively registered.

## Background

Retinal vein occlusion (RVO) is the second most common retinal vascular disease causing significant visual impairment in middle-aged and older adults, surpassed only by diabetic retinopathy. The choroid is composed of a matrix and multiple layers of blood vessels, which supply the outer structure of the retina and are important tissue structures with abundant blood flow in the eye. In recent years, an increasing number of studies have found that the choroid is related to the occurrence and development of many retinal diseases. Therefore, the use of choroidal imaging to study the pathogenesis of various retinal diseases has attracted more and more attention from scholars [1]. In clinical practice, frequency domain OCT enhanced depth imaging (EDI-OCT) technology is commonly used to present choroidal images and for various analyses. Most scholars use subfoveal choroidal thickness (SFCT) to evaluate the choroid, but SFCT varies greatly among different populations [2]. Therefore, in recent years, people have begun to explore the use of choroidal vascularity index (CVI) as a research indicator for choroidal observation. It can reflect changes in choroidal vascular composition in different diseases and also reflect the subsequent treatment effects of different diseases. At present, the choroidal vascularity index has been widely used in clinical observation. Previous literature has focused on the impact of common thick choroidal spectrum diseases or diabetes retinopathy on choroidal indicators and the changes of choroidal structure caused by fundus laser treatment of these fundus diseases [3–5], while there are few relevant reports on the changes of choroidal thickness and CVI in RVO patients after multiple anti VEGF treatments. This study adopts a retrospective research method to compare the differences in choroidal thickness and CVI at baseline between BRVO affected eyes and contralateral eyes. It also specifically observes the changes in choroidal thickness and CVI after multiple anti VEGF treatments in acute phase BRVO patients, as well as the changes in choroidal thickness and CVI during stable phase BRVO after macular edema subsides and treatment is stopped for at least six months. The aim is to explore the dynamic changes in choroidal indicators between acute and stable phases of BRVO and provide more detailed reference data for clinical research on CVI.

## Data and methods

### General information

Retrospective cohort study. A total of 91 patients with acute BRVO who underwent intravitreal injection of ranibizumab (0.5 mg/0.05 ml) at our intraocular injection center from January 2022 to June 2023 were selected. Among them, there were 44 males and 47 females with an average age of 59.15 ± 11.39 years. According to the classification of macular edema types, there were 18 cases of DRT, 30 cases of CME, and 43 cases of MIX. All enrolled cases were selected for monocular injection and followed up with 3 + PRN (pro re nata) treatment. This study complies with the Helsinki Declaration and has been approved by the hospital’s ethics review committee (approval number: 2023-145-001). Trial registration: ChiCTR, ChiCTR2400090054. Registered 13 November 2023.

### Inclusion and exclusion criteria

Inclusion criteria: (1) Clinically diagnosed monocular RVO with acute onset (onset not exceeding 3 months) and clinical symptoms of visual impairment due to macular edema, requiring intravitreal injection of anti VEGF drugs for treatment. (2) The central retina thickness measured by OCT during the first visit was greater than 300 μm.

Exclusion criteria: (1) Macular edema caused by other diseases. (2) Spherical equivalent refraction of ≥ ± 6.0 D or anisometropia. (3) Axial length > 26 mm or < 22 mm. (4) Unclear EDI-OCT choroidal imaging (such as vitreous hemorrhage, severe cataracts, and partial acute RVO due to severe retinal hemorrhage or high macular edema obscuring the underlying choroidal signal). (5) Eye or serious systemic diseases may affect choroidal blood flow (such as diabetes). (6) Previously received any intraocular injection therapy, retinal laser photocoagulation therapy, or intraocular surgery. (7) Serious systemic diseases such as cardiovascular and cerebrovascular diseases, as well as contraindications for local surgery.

### Inspection content

The content of eye examination includes best corrected visual acuity (BCVA), intraocular pressure (TOPCON), slit lamp examination, fundus examination, and fundus photography examination (ZEISS). Use SD-OCT (Heidelberg) for optical coherence tomography to confirm the diagnosis and classification of macular edema, use EDI-OCT mode for choroidal imaging, and conduct postoperative follow-up to monitor and evaluate treatment efficacy. BCVA examination was performed using a standard logarithmic visual acuity chart and converted to Logarithm of minimum angle of resolution (LogMAR) for statistical analysis. Record preoperative baseline and data of various indicators at least one month after each injection (as acute phase observation indicators), as well as data of various indicators at least six months after edema subsides and the condition stabilizes and medication is stopped (as stable phase observation indicators). Among them, 45 patients have complete acute and stable phase data.

### Classification of macular edema

**Fig. 1:**
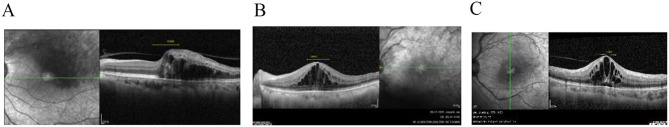
**A** Diffuse type (DRT), **B** Cystic edema type (CME), **C** Mixed type (MIX)

According to the OCT examination results, the morphological characteristics of macular edema are divided into three types: Type I: diffuse retinal thickening (DRT), characterized by diffuse sponge like retinal edema in the macular area and uniform reduction of internal retinal reflex (Fig. 1A); Type II: Cystoid macular edema (CME), characterized by one or more low reflex cysts visible in the retina of the macular area (Fig. 1B); Type III: Mixed type (MIX): Serous retinal detachment (SRD) with CME, defined as the presence of a low reflex space (SRD) between the retinal pigment epithelium and the neural sensory layer, as well as a low reflex capsule (CME) in the macular area (Fig. 1C). At baseline, according to the type of macular edema, there were 18 cases of DRT, 30 cases of CME, and 43 cases of MIX.

### Measurement and recording

The same experienced OCT operator performed the examination and independently measured the choroidal thickness of all patients. Use embedded tools to record the central retinal thickness (CRT) of all patients, and use the instrument software’s built-in caliper to measure the high reflection layer extending from the bottom of the outer edge of the retinal pigment epithelium to the choroidal sclera boundary as the choroidal thickness. Measure three locations per eye, namely the fovea centralis (SFCT), 1500 μm from the nasal side of the fovea centralis (CT N1.5 mm), and 1500 μm from the temporal side of the fovea centralis (CT T1.5 mm). Then calculate the average of the three locations (CT Mean) as the average choroidal thickness in the central macular area, and have the same experienced personnel process the image using Image J software to calculate the LA, SA, and TCA of the choroid within a range of 1500 μm from the fovea centralis. And CVI value (CVI = LA/TCA). A new item was added in “OCT evaluation” as follows: “ELM/EZ integrity score: 0 = intact, 1 = partial defect, 2 = extensive defect.

### Image J software image processing method [6]

**Fig. 2:**
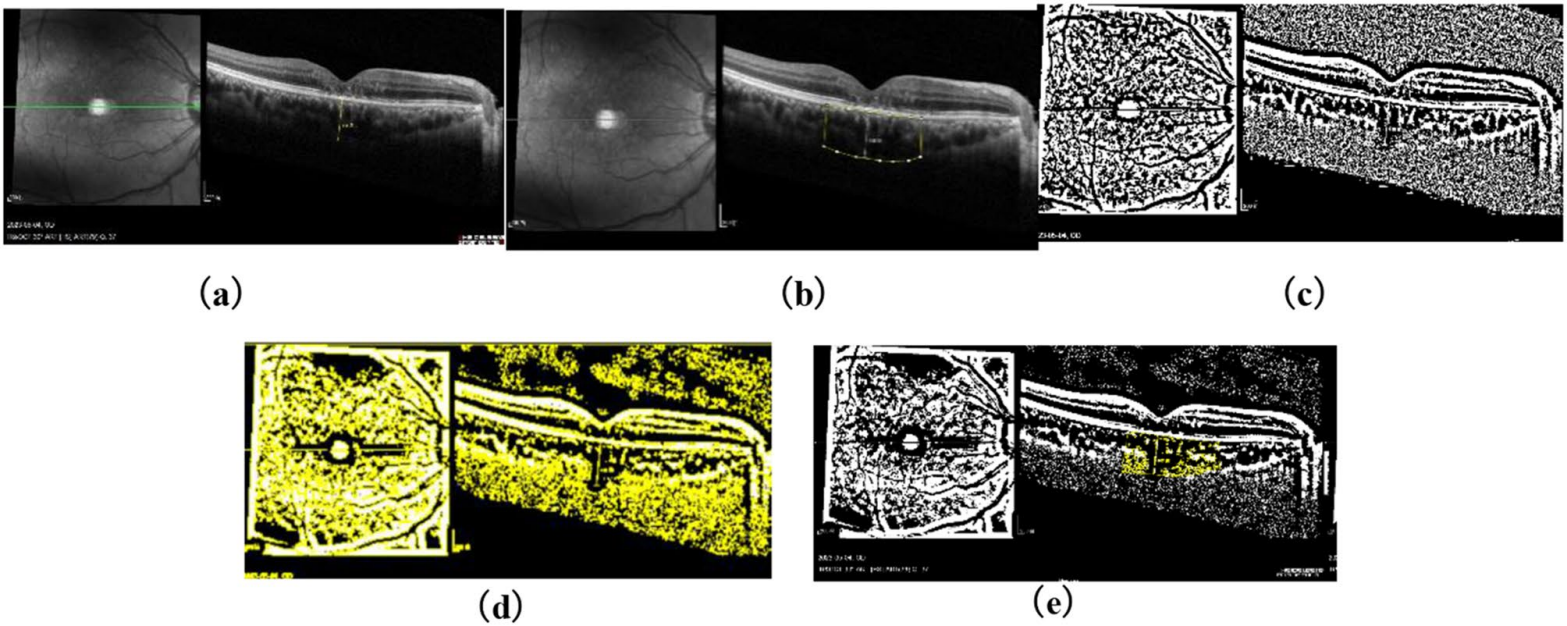
Shows an example of image binarization: (**a**) SD-OCT image (EDI mode), (**b**) polygon selection of the choroid below the 1500 μm horizontal line centered on the macular fovea, (**c**) image binarization using the Niblack method, (**d**) color threshold to highlight vascular areas; (**e**) The final result obtained by overlaying the luminal area measured in the 1500 μm horizontal line centered on the fovea on the SD-OCT image

The EDI-OCT image will be processed by the same image processing personnel. Use the polygon tool to mark the choroidal vascular area, and use the selection manager to record the selection. Remove the correspondence between pixels and actual size, and draw circles multiple times at the location representing the choroidal vascular lumen (lumen center) (sampling range 13px × 13px) to measure the grayscale values of four sampling points. Adjust the average grayscale value to make the dark parts of the image darker. Change the image type to 8-bit and use Niblack to automatically adjust the local threshold. Perform binary processing on the choroidal region in OCT scanning, then convert each binary image to RGB format, and use color thresholding tools to highlight the optical cavity region. Then calculate the total choroidal area, choroidal lumen area, and choroidal matrix area within the 1500 µ m central area of the macula. Bright pixels were defined as the choroidal stroma, while dark pixels were defined as the choroidal luminal area (See Fig. 2).

### Treatment methods

After thoroughly cleaning the conjunctival sac, use Alcon hydrochloride eye drops, 1 drop/5 minutes, for 3 consecutive times. Disinfect, lay towels, soak the conjunctival sac in 0.05% povidone iodine (Li Ercon, Shandong) for 1 min and 30 s, inject the drug (ranibizumab 0.5 mg/0.05 ml) into the vitreous cavity with a 30G needle 3.5–4 mm behind the corneal edge, evaluate the light perception after injection, and complete the surgery. All patients were treated with Levofloxacin Hydrochloride (Cravit, Santen) eye drops after surgery, 4 times a day for 7 consecutive days. All enrolled cases followed up with 3 + PRN (pro re nata) treatment. PRN retreatment criteria (retreatment triggered if ≥ 1 criterion met). (a) CRT ≥ 300 μm and an increase of ≥ 50 μm from the previous visit; (b) New or worsening intraretinal or subretinal fluid on OCT; (c) BCVA loss ≥ 0.2 LogMAR attributable to recurrent macular edema; (d) Evidence of active leakage on clinical examination or OCT angiography.

### Statistical analysis

Using SPSS 22.0 software, the quantitative data in this study followed a normal distribution through Shapiro Wilk normality test, expressed as mean ± standard deviation. The comparison of count data between groups was performed using a chi square test; Paired t-test was used to compare various indicators between the affected eye and the contralateral eye. The quantitative data of patients with different types of macular edema were compared between multiple groups using one-way analysis of variance. Repeated measures analysis of variance was used to compare various indicators within different time points before and after surgery, and LSD-t test was used for multiple comparisons. *P* ≤ 0.05 indicates that the difference is statistically significant.

### Sample size justification

During the design phase, we performed a power analysis for one-way repeated-measures ANOVA using PASS 22.0. Significance level α = 0.05; effect size f = 0.25 (medium); five repeated measures (baseline, after injections 1–3, and ≥ 6 months off treatment). The calculation indicated that a minimum of 42 eyes was required to detect the main effect of time on CVI with 90% power. We enrolled 91 patients and 45 patients had complete data (≥ 6 months after the last injection), which meets the requirement for the primary longitudinal analysis. Also, this was a retrospective paired design (affected vs. fellow eye). A paired t-test power analysis was conducted assuming a CVI difference of 3% and a pooled standard deviation of 4%. At α = 0.05 and 80% power, 22 pairs were required, we enrolled 30 available fellow-eye OCT datasets.

## Result

### Comparison of various indicators of different types of macular edema at baseline

At baseline, there was no statistically significant difference in the average age, gender, and eye type of patients with different types of macular edema. The MIX group had the highest proportion of grade 2 (60.5%) while CME group had the lowest proportion of grade 2 (13.3%) (see Table 1).

**Table 1:** Basic preoperative information of patients with different types of macular edema

Index	DRT(*n* = 18)	CME(*n* = 30)	MIX(*n* = 43)	F/χ^2^	*P*
Age (years)	57.94 ± 8.65	60.27 ± 11.36	58.88 ± 12.53	0.252	0.78
Gender Male (%)	12(27.3%)	14(31.8%)	18(40.9%)	3.178	0.20
Right Eye, n (%)	13(22.0%)	20(33.9%)	26(44.1%)	0.835	0.66
Grade 2 in ELM/EZ Distribution	9(50%)	4(13.3%)	26(60.5%)	22.735	< 0.01*

Note: *P* < 0.01 was considered statistically significant. DRT = diffuse retinal thickening; CME = cystoid macular edema; MIX = mixed type. ELM = External Limiting Membrane; EZ = Ellipsoid Zone

There was no statistically significant difference in BCVA and CVI between different types of macular edema. The CRT, CT N1.5 mm, average choroidal thickness, and SA in the CME group were lower than those in the DRT and mixed types, and the differences were statistically significant (*P* < 0.01) (see Table 2).

**Table 2:** Comparison of baseline indicators in patients with different types of macular edema

Index	DRT(*n* = 18)	CME(*n* = 30)	MIX(*n* = 43)	F	*p*
BCVA (LogMar)	0.85 ± 0.49	0.78 ± 0.29	0.82 ± 0.34	0.252	0.78
CRT(um)	601.50 ± 176.97	519.77 ± 166.49 ^b^	651.12 ± 195.91	4.554	0.01*
SFCT (um)	346.44 ± 72.10	292.67 ± 62.90	327.26 ± 90.60	2.992	0.055
CT N1.5 mm(um)	307.50 ± 86.88	249.80 ± 50.56 ^a^	286.65 ± 94.91	3.235	0.04*
CT T1.5 mm(um)	321.78 ± 85.29	280.10 ± 47.59	301.12 ± 74.63	2.067	0.13
CT Mean(um)	325.24 ± 77.32	274.19 ± 45.48 ^a^	307.02 ± 82.45	3.303	0.04*
LA(mm^2^)	1.45 ± 0.36	1.38 ± 0.42	1.52 ± 0.44	0.910	0.41
SA(mm^2^)	0.57 ± 0.20	0.48 ± 0.18	0.62 ± 0.^21b^	4.258	0.02*
TCA(mm^2^)	2.02 ± 0.46	1.86 ± 0.56 ^b^	2.13 ± 0.58	2.064	0.13
CVI(%)	71.73 ± 6.58	74.33 ± 5.28	71.19 ± 5.69	2.767	0.07

Note: *P* < 0.05 was considered statistically significant. a Compared with DRT group, *P* < 0.05. b Compared with CME group, *P* < 0.05

Data are presented as mean ± standard deviation. DRT = diffuse retinal thickening; CME = cystoid macular edema; MIX = mixed type; BCVA = best-corrected visual acuity; CRT = central retinal thickness; SFCT = subfoveal choroidal thickness; CT N1.5 mm = nasal choroidal thickness 1.5 mm from fovea; CT T1.5 mm = temporal choroidal thickness 1.5 mm from fovea; CT Mean = mean choroidal thickness; LA = luminal area; SA = stromal area; TCA = total choroidal area; CVI = choroidal vascularity index

### Comparison of various indicators between the affected eye and the contralateral eye at baseline

Comparing the BRVO affected eye with the contralateral eye, the BCVA of the affected eye was worse than that of the contralateral eye, and the CRT was thicker than that of the contralateral eye, with significant statistical significance (*P* < 0.01). The SFCT, CT T1.5 mm, CT Mean, LA, SA, TCA, and CVI values of the affected eye were higher than those of the contralateral eye, and the differences were statistically significant (*P* < 0.01) (see Table 3).

**Table 3:** Comparison of baseline indicators between the affected eye and the contralateral eye

Index	Affected eyes (*n* = 30)	Opposite eye (*n* = 30)	t	*P*
BCVA(LogMar)	0.76 ± 0.34	0.92 ± 0.13	9.846	< 0.01**
CRT(um)	594.21 ± 198.62	225.48 ± 10.85	10.119	< 0.01**
SFCT(um)	327.21 ± 77.24	245.21 ± 36.20	6.65	< 0.01**
CT N1.5 mm(um)	271.83 ± 71.08	230.48 ± 25.69	3.147	< 0.01**
CT T1.5 mm(um)	296.03 ± 59.97	233.76 ± 3.92	5.100	< 0.01**
CT Mean(um)	298.36 ± 59.98	236.48 ± 29.63	5.817	< 0.01**
LA(mm^2^)	1.53 ± 043	0.91 ± 0.21	10.391	< 0.01**
SA(mm^2^)	0.63 ± 0.21	0.43 ± 0.14	5.530	< 0.01**
TCA^(mm2^)	2.16 ± 0.60	1.33 ± 0.30	9.594	< 0.01**
CVI(%)	70.99 ± 4.40	68.28 ± 6.33	1.945	< 0.01**

Note: *P* ≤ 0.01 was considered statistically significant. Data are presented as mean ± standard deviation. BCVA = best-corrected visual acuity; CRT = central retinal thickness; SFCT = subfoveal choroidal thickness; CT N1.5 mm = nasal choroidal thickness 1.5 mm from fovea; CT T1.5 mm = temporal choroidal thickness 1.5 mm from fovea; CT Mean = mean choroidal thickness; LA = luminal area; SA = stromal area; TCA = total choroidal area; CVI = choroidal vascularity index

### Dynamic changes of various indicators after multiple injections in the surgical eye

Compare the level changes of various indicators at different time points between the stable and acute phases of the surgical eye that has undergone multiple vitreous injections and stopped treatment for at least six months. Patients with BRVO showed significant improvement in BCVA and CRT after multiple injections in the affected eye, with statistical significance (*P* < 0.01). After stopping the injection for six months, BCVA further improved, while CRT remained relatively stable compared to the last injection. The choroidal thickness (SFCT, CT N1.5 mm, CT T1.5 mm, CT Mean) gradually decreased from baseline after the first injection, and the difference was statistically significant (*P* < 0.01). It remained relatively stable after the second injection and remained stable at least six months after the last injection was stopped. LA, SA, and TCA gradually decreased compared to baseline after anti-VEGF injection, with statistically significant differences (*P* < 0.01), and their decreasing trend continued until the last injection was stopped for at least six months. CVI significantly decreased after the second injection and remained stable thereafter until at least six months after the last injection was stopped, with a statistically significant difference (*P* < 0.01) (see Table 4).

**Table 4:** Changes in various indicators between the acute and stable phases of BRVO

Index	Timepoint				At least 6 months after the last injection	F	*P*
	Baseline	After the first surgery	After the second surgery	After the third surgery			
BCVA(LogMar)	0.78 ± 0.35	0.42 ± 0.25^a^	0.29 ± 0.20^ab^	0.22 ± 0.17^abc^	0.18 ± 0.12^abcd^	111.275	< 0.01**
CRT(um)	595.96 ± 153.87	287.51 ± 106.23^a^	234.91 ± 66.54^ab^	229.13 ± 45.69^ab^	245.47 ± 54.74^a^	136.209	< 0.01**
SFCT(um)	323.20 ± 84.69	272.27 ± 63.60^a^	247.51 ± 52.35^ab^	250.80 ± 56.08^a^	243.78 ± 64.44^ab^	26.515	< 0.01**
CT N1.5 mm(um)	280.69 ± 73.54	265.69 ± 76.20^ab^	240.36 ± 55.41^ab^	240.91 ± 67.88^ab^	220.47 ± 65.21^ab^	15.885	< 0.01**
CT T1.5 mm(um)	311.87 ± 68.61	276.47 ± 55.11^a^	247.44 ± 51.05^ab^	244.71 ± 55.81^ab^	254.24 ± 54.63^a^	22.009	< 0.01**
CT Mean(um)	305.25 ± 68.04	271.47 ± 56.39^a^	245.10 ± 45.83^ab^	245.47 ± 51.92^ab^	239.49 ± 51.53^ab^	37.993	< 0.01**
SA(mm^2^)	0.56 ± 0.19	0.39 ± 0.11^a^	0.40 ± 0.11^ab^	0.40 ± 0.11^ac^	0.32 ± 0.06^abd^	41.038	< 0.01**
LA(mm^2^)	1.48 ± 0.45	1.08 ± 0.28^a^	0.96 ± 0.17^ab^	0.97 ± 0.26^a^	0.78 ± 0.20^abcd^	68.958	< 0.01**
TCA(mm^2^)	2.04 ± 0.59	1.47 ± 0.35^a^	1.36 ± 0.22^a^	1.37 ± 0.33^a^	1.10 ± 0.24^abcd^	79.682	< 0.01**
CVI(%)	72.34 ± 4.67	72.10 ± 4.85	70.51 ± 5.71	70.46 ± 6.01	70.14 ± 4.04^b^	4.008	< 0.01**

Note: *P* < 0.01 was considered statistically significant. a Compared with Baseline, *P* < 0.01. b Compared with the first injection, *P* < 0.01. c Compared with the second injection, *P* < 0.01. d Compared with the third injection, *P* < 0.01. Data are presented as mean ± standard deviation. DRT = diffuse retinal thickening; CME = cystoid macular edema; MIX = mixed type; BCVA = best-corrected visual acuity; CRT = central retinal thickness; SFCT = subfoveal choroidal thickness; CT N1.5 mm = nasal choroidal thickness 1.5 mm from fovea; CT T1.5 mm = temporal choroidal thickness 1.5 mm from fovea; CT Mean = mean choroidal thickness; LA = luminal area; SA = stromal area; TCA = total choroidal area; CVI = choroidal vascularity index

### Number of injections

Overall, the 91 patients received a mean of 4.6 ± 1.3 intravitreal ranibizumab injections (range 3–8). Among the 45 patients who completed both acute and stable-phase follow-up, the mean was 4.6 ± 1.1 injections.

## Discussion

Retinal vein occlusion (RVO) is a common retinal vascular disease that causes a significant decline in the vision of middle-aged and elderly people. The main risk factors include hypertension, diabetes, atherosclerosis, hyperlipidemia, hyperviscosity, coagulation dysfunction, glaucoma, etc [7]. The occurrence of RVO is caused by various reasons leading to endothelial damage of the vascular wall, increased levels of various cytokines and inflammatory factors, which disrupt the retinal barrier and lead to macular edema. The most critical factor in the pathogenesis of the disease is vascular endothelial growth factor (VEGF). Therefore, intravitreal injection of anti VEGF drugs is currently considered the first-line treatment for RVO macular edema.

Optical coherence tomography (OCT) is a non-invasive imaging tool that can be used for in vivo detection of retinal and choroidal structures. Enhanced depth imaging (EDI) mode can display deep choroidal layers with higher clarity. Many scholars use EDI-OCT to observe changes in choroidal thickness in RVO patients. Hae Min Kang et al. [8] reported that in eyes affected by RVO, the choroidal thickness around the optic disc and the fovea centralis was significantly reduced after 12 months of anti VEGF treatment. Muge Coban Karatas et al. [9] reported that SFCT in RVO patients showed significant thickening compared to the contralateral eye, and SFCT decreased significantly after anti VEGF treatment. Some scholars have also suggested that there is a significant difference in SFCT between RVO eyes and contralateral eyes [10], but SFCT did not decrease after anti VEGF treatment [11]. It can be seen that these reports on the choroidal thickness of RVO eyes and the changes in choroidal thickness after treatment are not completely consistent, suggesting that the factors affecting choroidal thickness may not be singular.

The choroid is the largest vascular rich tissue structure in the human eye, with choroidal blood flow accounting for 85% of all eye blood flow [12]. Therefore, due to changes in hydrostatic pressure and VEGF levels in RVO patients, in addition to causing retinal lesions, the lesions may also involve the choroid. The Choroidal Vascularity Index (CVI) originated from Sonoda et al.‘s study, which binarized the images of the choroid under EDI-OCT. Black represents luminal area (LA), white represents stromal area (SA), and the sum of the two is the total choroidal area (TCA). LA/TCA is used to represent choroidal blood flow perfusion [13], and it is named the Choroidal Vascularity Index (CVI) [2]. Due to its independence from age, refractive errors, axial length, and intraocular pressure, CVI is considered a relatively effective tool for evaluating choroidal vascular changes and has been used in research on various eye diseases such as CSC, AMD, DME, etc [14–17].

According to the morphological characteristics of OCT, patients with macular edema can be further classified into four types: DRT, CME, SRD, and mixed type [18]. In the cases we studied, we have not yet found a simple SRD type, and patients with SRD are all accompanied by CME. Therefore, in this study, patients were classified into DRT, CME, and mixed type (SRD combined with CME) according to the morphology of macular edema. Our research results show that the CRT and SFCT of the CME group are lower than those of the mixed type, which is consistent with the research results of Chen Lulu et al. [19]. They defined the SRD group as the SRD group, which is the mixed type group in our study. They found that when CRVO and BRVO patients develop the disease, the choroidal thickness (corresponding to our mixed type) of the SRD group is thicker than that of the simple CME group, which is consistent with our research results. At the same time, some scholars have found that the BCVA of patients with mixed type ME is significantly worse. We also found that the BCVA of patients with mixed type macular edema is lower than that of the CME group, which may be because mixed type is further developed on the basis of SRD and CME. At this time, the outer membrane and ellipsoidal zone of the macular area in patients are mostly damaged, resulting in poorer vision. We added “OCT evaluation” as follows: ELM/EZ integrity score: 0 = intact, 1 = partial defect, 2 = extensive defect. ELM/EZ defect scores were significantly higher in MIX group than in CME group, BCVA in MIX group was poorer than that in CME group, suggesting that outer retinal damage is an important factor in visual impairment. This is generally consistent with the analysis in the literature [20, 21]. At the same time, we found that the SA and TCA values of the CME group were smaller than those of the other two groups, but there was no statistically significant difference in CVI among the three groups.

Meanwhile, we also compared the BRVO affected eye with the contralateral eye. As this section is a retrospective analysis, only 30 patients underwent binocular OCT examination at baseline in our data. Therefore, we compared the baseline indicators of the affected eye and the contralateral eye in these 30 patients, which meets the minimum sample size requirement in statistics. In these patients, we observed that the choroidal thickness of the affected eye was significantly thicker than that of the contralateral eye. During acute onset of BRVO, the choroidal vascular lumen area and choroidal matrix area increased compared to the contralateral unobstructed eye, and the difference was statistically significant, which is consistent with the research findings of Tang F et al. [22]. We know that acute onset of RVO leads to elevated levels of VEGF in the eye, which in turn causes secondary choroidal edema. The elevation of VEGF stimulates the production of nitric oxide, vasodilation, and increased ocular blood flow, Elevated VEGF is the main cause of increased vascular permeability and choroidal thickness in the choroid layer [23, 24], but some scholars have come to different conclusions, which may be due to the different stages of onset of RVO included in the study. For example, Aribas YK et al. found [25] that there was no statistical difference in the central choroidal thickness of retinal vein occlusion eyes compared to the control group. The OCT scan they analyzed was obtained after treatment of macular edema, which may have missed the acute phase of choroidal thickening. Therefore, we were unable to find a significant difference in the thickness of the central depression choroid between the RVO group and the control group. Our study focused on BRVO affected eyes in the acute phase, and we found that the choroidal thickness of BRVO affected eyes in the acute phase was not significantly different. The thickness should be higher than the unobstructed eye on the opposite side. We also found that the LA, SA, TCA, and CVI values of the BRVO affected eye were higher than those of the contralateral eye, and the differences were statistically significant. We know that the choroid is regulated by the autonomic and sensory nerves. Therefore, in acute RVO, choroidal thickening may be due to a compensatory increase in choroidal blood flow caused by local autoregulatory muscle generation mechanisms (related to vascular smooth muscle) due to retinal ischemia, which compensates for the decrease in retinal blood vessels and leads to choroidal thickening. At the same time, due to the increase in VEGF levels in the eye, choroidal vascular permeability increases and passively swells and thickens. Therefore, choroidal thickening in BRVO patients is secondary to retinal ischemia rather than primary choroidal thickening.

We adopted a 3 + PRN treatment method for all patients, and after 3 injections, BCVA and CRT in the affected eye improved significantly compared to before. Choroidal thickness, LA, SA, TCA, and CVI decreased after the first and second injections, and remained relatively stable after the second injection. We speculate that the main reason for the rapid thinning of choroidal thickness after intravitreal injection of ranibizumab is that ranibizumab can penetrate through the retina and into the choroid, improving the retinal barrier function and reducing the permeability of retinal capillaries, while also reducing the permeability of choroidal blood vessels, thereby reducing choroidal thickness.

After repeated anti VEGF treatment in the acute phase of BRVO, macular edema disappears. Over time, collateral circulation gradually opens up and enters a stable phase. We retrospectively reviewed data from 45 patients with stable RVO who had macular edema resolution and had their last injection stopped for at least six months. We analyzed their choroidal parameters and observed the dynamic changes in choroidal parameters after multiple treatments with ranibizumab during acute onset of BRVO and at least six months after stopping injection during stable BRVO. We found that after stopping injection and entering a stable phase, the CRT of BRVO patients remained relatively stable compared to the last injection, but the BCVA further increased compared to the last injection, indicating that many visual cell functions that were previously damaged by macular edema still have room for further improvement and recovery after the edema subsides. The choroidal thickness gradually decreased after anti VEGF treatment and remained relatively stable thereafter; LA, SA, TCA, and CVI gradually decreased after anti VEGF injection, and the downward trend continued until the last injection was stopped for at least six months. It can be seen that after the acute phase treatment of BRVO, the level of VEGF in the eye decreases, the leakage of choroidal blood vessels decreases, and the choroidal blood vessels are further reshaped.

Although ranibizumab has a half-life of only 9 days, repeated dosing can induce cumulative “VEGF signaling silencing” in choroidal capillary endothelial cells, this promotes pericyte reattachment, basement membrane thickening, and lumen retraction, resulting in the continuous reduction of LA ([26]. Meanwhile, the activity of extracellular matrix (ECM) degradation enzymes/matrix metalloproteinases (MMP-2/-9) is reduced, leading to decreased collagen deposition, which explains the synchronous decline in SA and TCA [27]. During the acute phase of BRVO, sympathetic nerve stimulation induced by ischemia gradually weakens in the stable phase. Choroidal blood flow autoregulation shifts from “compensatory hyperperfusion” to “baseline downregulation,” further promoting vasoconstriction and stromal retraction, which is consistent with the continued decline in CVI.” Thus, we believe that the sustained decline in LA/SA/TCA/CVI supports a combined mechanism of “chronic vascular remodeling” rather than a single drug residual effect. Future studies will quantify choroidal capillary density using OCTA to verify the above hypotheses.

In this study, choosing CVI as our research indicator has certain advantages, as it is less affected by physiological factors and has less variability compared to traditional choroidal thickness. However, it still has some limitations in clinical use. One is to obtain high-quality and clear EDI-OCT scan images, otherwise the reliability of CVI is poor. Secondly, the measurement area boundary of CVI needs to be manually drawn, which is inevitably affected by subjective factors. Thirdly, the EDI-OCT images obtained need to be binarized using Image J software, which is a relatively complex and time-consuming process. To avoid the limitations mentioned above, we excluded all OCT images with unclear choroidal imaging. In addition to excluding images caused by severe cataracts, vitreous hemorrhage, etc., we also excluded images with unclear choroidal imaging due to excessive RVO retinal hemorrhage or high macular edema in the acute phase. All images were manually drawn by the same experienced physician to measure the boundaries of the measurement area, in order to ensure the accuracy of the data in this study and reduce bias caused by human factors. Although CVI is considered to be less affected by physiological factors such as age, refractive status, and intraocular pressure, recent studies have suggested that axial length may have some impact on CVI values [28]. To minimize the potential bias caused by this factor, our study excluded patients with an axial length > 26 mm or < 22 mm in the inclusion criteria. However, we acknowledge that this does not completely eliminate the potential impact of axial length on CVI. Future studies will further control for confounding factors such as axial length through subgroup analysis or multivariate regression models to more accurately assess the changes in CVI in BRVO patients.

In addition to measuring the thickness of the choroid in the fovea centralis, our study also measured the choroid thickness at a distance of 1500 μm from the nasal and temporal sides of the fovea centralis to calculate the average choroid thickness in the central area of the macula. Multi point measurement can improve the accuracy of choroid thickness. At the same time, we used the CVI index to evaluate the structure and morphology of the choroid, which can greatly reduce bias caused by choroid thickness variation. A small amount of literature has reported changes in choroidal thickness in RVO patients after anti VEGF treatment, mostly as a result of a single injection, and the results are not consistent. We compared the choroidal thickness and CVI in different types of RVO-ME, and also compared the dynamic changes in choroidal thickness and CVI in BRVO after multiple anti VEGF treatments, as well as in the acute and stable phases. This provides new insights for evaluating the changes in choroidal vessels caused by different stages of BRVO lesions.

Although we have chosen the same doctor to measure choroidal thickness, there is always potential bias in manually measuring choroidal thickness, and choroidal thickness exhibits diurnal variation. A limitation of this retrospective study is that OCT measurements were not performed at the same time of day for all patients, which may result in some bias. At the same time, the thickness of the choroid is affected by many factors such as age, time, and eye axis, and it is difficult to ensure completely consistent measurement conditions, which are our limitations. When we enrolled, we excluded patients with refractive error equivalent spherical lens ≥ ± 6.0D and axial length > 26 mm or < 22 mm to minimize choroidal measurement bias. At the same time, we introduced the choroidal vascularity index (CVI) to evaluate choroidal blood flow perfusion, in order to compensate for the deficiency. Given the retrospective design, no OCTA or FFA examinations had been conducted previously, so we were unable to obtain data on ischemic or non-ischemic RVO. This is also our limitation. In future multi-center prospective studies, we will increase the observation of the impact of ischemia on choroid.And it should be noted that, although fellow eyes displayed no obvious abnormalities on routine fundus examination or OCT, subclinical choroidal haemodynamic changes may still exist. Thus, the observed ‘affected-eye vs fellow-eye’ differences in the present study may be marginally amplified by systemic factors. Future studies will incorporate OCTA to quantify the potential impact of systemic factors on fellow eyes.

## Conclusion

Retinal vein branch occlusion not only affects the structure of the retina, but also has an impact on the morphology of the choroid and blood flow perfusion. The choroidal vasodilation, choroidal matrix thickening, choroidal thickness, and CVI of retinal vein occlusion eyes are higher than those of non-occluded eyes, and the CVI value does not vary depending on the type of BRVO macular edema. During the acute phase, BRVO anti VEGF treatment can reduce choroidal thickness and CVI, gradually entering a stable phase.

## Data Availability

Data and materials can be asked from the correspondence author by Email.

## Abbreviations

BRVO: Branch Retinal Vein Occlusion
CRT: Central Retina Thickness
SFCT: Subfoveal Choroidal Thickness
CT N1.5mm: Nasal Choroidal Thickness at 1.5 mm of macula
CT T1.5mm: Temporal Choroidal Thickness at 1.5 mm of macula
CT mean: Mean Macular Thickness
LA: Luminal Area
SA: Stromal Area
TCA: Total Choroidal Area
CVI: Choroidal vascularity index
EDI: Enhanced Depth Imaging
anti-VEGF: anti-Vascular Endothelial Growth Factor
BCVA: Best-Corrected Visual Acuity
OCT: Optical Coherence Tomography
DRT: Diffuse Retinal Thickening
CME: Cystoid Macular Edema
SRD: Serous Retinal Detachment
